# Temperature profile characterization with fluorescence lifetime imaging microscopy in a thermophoretic chip

**DOI:** 10.1140/epje/s10189-021-00133-7

**Published:** 2021-10-19

**Authors:** Namkyu Lee, Dzmitry Afanasenkau, Philipp Rinklin, Bernhard Wolfrum, Simone Wiegand

**Affiliations:** 1 IBI-4:Biomacromolecular Systems and Processes, Forschungszentrum Jülich GmbH, D-52428 Jülich, Germany; 2 Technische Universität Dresden Center for Molecular and Cellular Bioengineering, D-01062 Dresden, Germany; 3 Neuroelectronics, Munich School of Bioengineering, Department of Electrical and Computer Engineering, Technical University of Munich, D-85748 Garching bei München, Germany; 4 Chemistry Department-Physical Chemistry, University Cologne, D-50939 Cologne, Germany

## Abstract

**Abstract:**

This study introduces a thermophoretic lab-on-a-chip device to measure the Soret coefficient. We use resistive heating of a microwire on the chip to induce a temperature gradient, which is measured by fluorescence lifetime imaging microscopy (FLIM). To verify the functionality of the device, we used dyed polystyrene particles with a diameter of 25 nm. A confocal microscope is utilized to monitor the concentration profile of colloidal particles in the temperature field. Based on the measured temperature and concentration differences, we calculate the corresponding Soret coefficient. The same particles have been recently investigated with thermal diffusion forced Rayleigh scattering (TDFRS) and we find that the obtained Soret coefficients agree with literature results. This chip offers a simple way to study the thermophoretic behavior of biological systems in multicomponent buffer solutions quantitatively, which are difficult to study with optical methods solely relying on the refractive index contrast.

**Graphic abstract:**

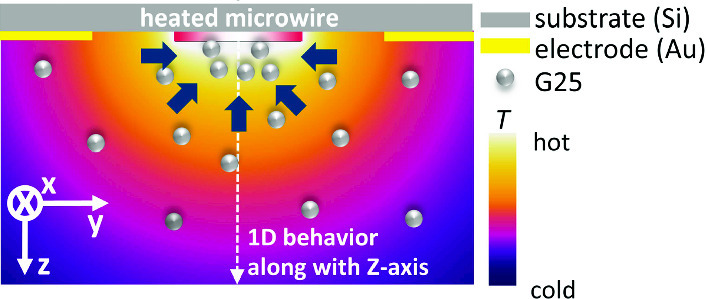

**Supplementary Information:**

The online version contains supplementary material available at 10.1140/epje/s10189-021-00133-7.

## Introduction

Thermophoresis is the mass transport caused by a temperature gradient. It gained a lot of interest in biotechnology due to its high sensitivity to changes in the hydration layer as they occur in biological systems during protein–ligand binding or conformational transitions. The main analytical method that uses thermophoresis is the MicroScale Thermophoresis (MST) [1], which monitors protein–ligand binding, changes in the hydrating layer and conformation. These results improved drug selection processes in pharmaceutical industry. Furthermore, it has been used to reveal disease mechanisms in biomedicines such as influenza [2], Alzheimer [3] and corona [4]. However, MST records only relative thermophoretic changes without quantifying thermophoretic coefficients such as the Soret coefficient, $S_\mathrm T$, describing the ratio of the established concentration $\nabla c$ and temperature gradient $\nabla T$ in the steady state [5]. $S_\mathrm {T}$ is very sensitive to various parameters such as size, pH and ionic strength in biological solution and can be used as a monitor to probe entropic and enthalpic changes in biological systems. Thus, for applying thermophoresis as an analytical method, an advanced microscale device is needed for measuring Soret coefficients quantitatively in systems containing proteins, ligands and buffer compounds.

Available methods for thermophoresis are thermal lens [6], thermal diffusion forced Rayleigh scattering (TDFRS) [7], beam deflection [8–10] and thermogravitational columns (TGC) [11]. Most experimental methods probe the concentration change optically by measuring the refractive index contrast. Since these methods are limited to measure thermophoretic properties of binary and specific ternary mixtures, they are not appropriate for proteins in buffer solutions. Another method to investigate ternary and multicomponent mixtures is classical TGCs, but these columns require sample volumes on the order of 30 mL [12], so that the technique is unsuitable for biological samples that are only available in small amounts of a few microliter.

Over the last decade, researchers have developed microscale devices to measure thermophoretic properties using small sample volumes in this range [9, 13–21]. Moreover, those devices have shorter equilibrium times due to smaller dimensions. Since the diffusion coefficient is inversely proportional to the size of colloids, the microscale range makes it possible to investigate (bio)colloids with a diameter of the order of ${\upmu }$m. Often the devices use direct observation of large colloids or fluorescently labeled smaller particles. Under these conditions, the thermophoretic motion of particles of interest can be determined even in the presence of other compounds, e.g., buffer components.

For the development and validation of theoretical models, which describe entropic and enthalpic changes during protein-ligand binding or conformational change of the protein, it is important to have a solid database. However, while general trends of microscale devices are often confirmed by other experiments [13, 15, 20, 22], a direct comparison of results of the same system with validated methods as done for low-molecular weight compounds remains challenging [23]. For instance, it turned out that thermophoretic measurements of a protein–ligand system with an established method are difficult to analyze as it is not always possible to separate signal contributions stemming from buffer, ligand and protein simultaneously [24]. Those difficulties could certainly be avoided, if the thermophoretic motion of a protein could be monitored directly using a thermophoretic chip.

On the other hand, a crucial point in microscale devices is the difficulty to characterize the temperature profile within the device [22]. Typically, temperature measurements are performed using a resistive temperature detector (RTD) or a thermocouple located near the measuring chamber. Often these measurement points are outside of the sample chamber, so that heat transfer equations are needed to obtain a complete temperature profile [22]. However, low-temperature gradients are easily affected by external conditions, which makes it necessary to measure the temperature distribution within the measurement cell instead of calculating the profile from temperature measurements at two points outside of the cell. For instance, fluorescence dyes can be used to measure the temperature distribution inside the measurement chamber as fluorescence intensity and lifetime depend on temperature. While the fluorescence intensity has been utilized for temperature measurements in thermophoretic devices [25], fluorescence lifetime imaging microscopy (FLIM) has so far not been used despite its robustness against the external environment. Thus, we utilize FLIM for analyzing the temperature distribution inside the measured chamber.

Herein, we introduce a thermophoretic chip for measuring the Soret coefficient using a confocal microscope. Applying a current through a microscopic conductor on the chip’s surface causes a temperature gradient around the wire. Since the temperature field at different heights is two-dimensional, we concentrate our analysis to areas for which a one-dimensional temperature gradient is predicted. With an applied resistive heating, a large temperature gradient $(10^{6})$ ${\mathrm {K/m}}$ can be achieved [22, 26], which induces a movement of particles either to the cold or to the warm side, depending on their thermophoretic properties. The investigated particles are polystyrene particles with a diameter of 25 nm containing a fluorescence dye inside. The results are compared to a recent study of the same particles using a validated optical method referred to as thermal diffusion forced Rayleigh scattering (TDFRS) [23, 27].

**Fig. 1:**
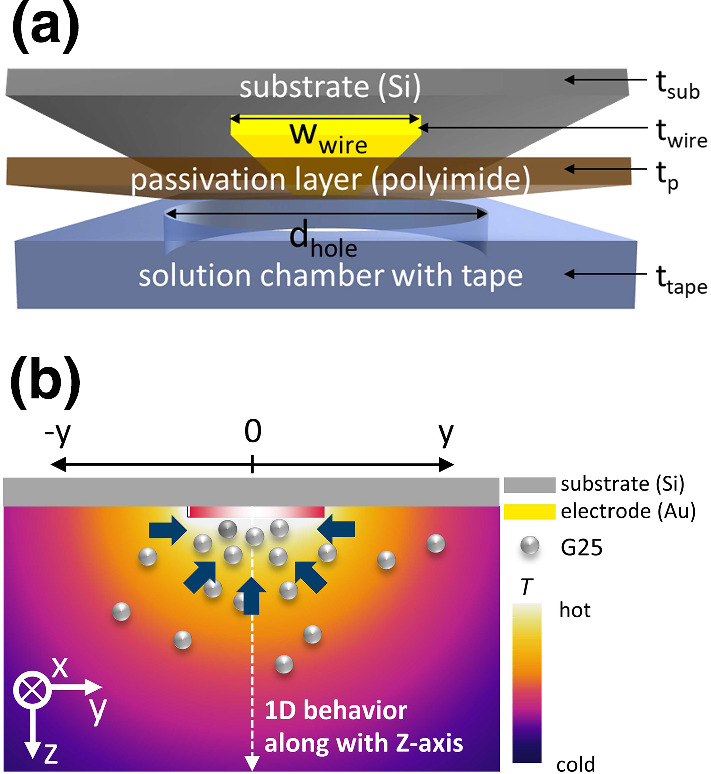
**a** Schematic of deposited layers of the thermophoretic chip in the *yz*-plane. **b** Illustration of the thermophoretic behavior around a heated microwire

## Experimental details

### Fabrication of the thermophoretic chip

Figure 1a shows a schematic of the deposited layers and a photograph of a fabricated chip. In general, more than one wire can be addressed on this multipurpose chip, but in this study we used only a single wire to create a temperature gradient. We prepared a thermally oxidized silicon wafer (Si-Mat Silicon Materials, Kaufering, Germany) with a thickness ($t_{\mathrm {sub}}$) of 500 $\upmu$m. The microwire pattern was created with photolithography utilizing a double-layer resist (LOR3B; Microchem, Newton, MA and NLOF2020; MicroChemicals, Ulm, Germany) and a developer (MIF 326, Microresist Technology, Berlin, Germany). The metal layer of Ti/Au/Ti (thickness ($t_{\mathrm {wire}}$), 10/400/10 nm) with Ti as adhesive layer, was deposited by sputter deposition (LLs Evo II, Oerlikon Systems, Balzers, Liechtenstein). The width of metal wire ($w_{\mathrm {wire}}$) was 10 $\upmu$m. On top of the microwire array, a 1.4 $\upmu$m polyimide layer ($t_{\mathrm {p}}$) (PI-2610, HD Microsystems GmbH, Germany) for a passivation layer is spin-coated (6000 rpm, 30 s). For opening the chip’s contact pads, photolithography (AZ-5340, AZ Electronics Materials GmbH, Wiesbaden, Germany) and reactive ion etching (RIE) were applied subsequently, and afterward, the chip was cleaned with acetone. The solution chamber was created using transparent double-sided tape ($t_{\mathrm {tape}}$ = 50 $\upmu$m) with a hole ($d_{\mathrm {hole}}$ = 3.5 mm) and a glass coverslip (180 $\upmu$m, Polysciences Inc. USA). For sealing, a grease (K.W.S. joint grease, Germany) was used to prevent leakage of the solution.

### Sample preparation

Polystyrene (PS) particles G25 (ThermoFisher Scientific Inc.) with a particle diameter of 25 nm were used without further treatment (run 1 and 2). The particle concentration was 1 wt% in a water based solution with a ${\mathrm {pH}} = 3.2$. Particles had a density of 1.05 ${\mathrm {g/cm}}^{3}$ and a refractive index of $n = 1.59$ at a wavelength of 589 nm at $T=25^{\circ }$C. Firefly fluorescence green dye was used inside the spherical particles. According to the information provided by the supplier, the suspension contained a trace amount of ethylenediaminetetraacetic acid (EDTA) and an anionic surfactant similar to sodium dodecyl sulfate (SDS) as a preservative to inhibit aggregation and promote stability [27]. Additionally, we investigated a washed solution (runs 3 and 4) with a particle concentration of 0.5 wt% containing 3 mM SDS for stabilization. A detailed description of the preparation can be found in the previous paper [27].

### Temperature and concentration measurement

In order to measure the temperature and concentration profiles, we utilized a confocal fluorescence microscope (Olympus IX71). The lateral and axial resolution are 0.2 $\upmu$m and 0.6 $\upmu$m, respectively. The chip was installed in the microscope in the reverse direction to suppress convection. Details of the setup are presented in the supporting information (Sec. S1). Using simulations, we compared the thermophoretic velocity with the free convection velocity. The free convection velocity ranges from 0.05 $\upmu$m/s to 0.16 $\upmu$m/s as a function of the distance of the wire. The thermophoretic velocity is 0.77 $\upmu$m/s [27]. This implies that the free convection velocity reaches 20% of the thermophoretic velocity far away from the wire. $S_\mathrm {T}$ obtained with the chip agrees with $S_\mathrm {T}$ determined with the TDFRS, meaning that the free convection is negligible. For this reason, we neglected the free convection as it is of the same order as our uncertainty. However, since the employed geometry will always lead to the free convection, it should be considered for systems with a lower thermal diffusion coefficient. Note that, if we know the exact boundary condition and the temperature distribution in the chamber, then the numerical simulation will be helpful for analyzing the influence due to the free convection on $S_\mathrm {T}$.

Each experiment is performed in two steps: first the temperature distribution is determined from a RhB solution, and then, the cell is cleaned thoroughly and refilled with the sample solution. This procedure typically results in a slight shift of the peak position of the concentration profile compared to the temperature scan, because it is impossible to reproduce the same location of the chip within 1 $\upmu$m by hand. However, to analyze the data, we superimpose the positions of the maxima of the temperature and concentration scans. We did not perform direct measurements in RhB solution to avoid interference of the dye with the thermophoresis of the colloids due to charge or preferential adsorption on the surface. Note that the temperature profile for temperature and concentration measurement is the same, since in both cases highly diluted aqueous solutions with almost identical thermal conductivity are used. If it is not possible to use the same solvent, care should be taken to ensure that they have similar thermal conductivities and viscosities.

For temperature measurements as a first step, we used the temperature sensitivity of the fluorescence lifetime of Rhodamine B (RhB, Sigma Aldrich, grade: for fluorescence). In order to measure the fluorescence lifetime image, we filled the sample chamber with a solution of 0.3 mg $\hbox {mL}^{-1}$ RhB dissolved in MilliQ water. The chamber was sealed with a vacuum grease (K.W.S. joint grease, Germany) and a glass cover slip. Using a calibration curve given in the supporting information (Sec. S2), we converted the measured fluorescence lifetime $\tau (x,y)$ into a temperature profile *T*(*x*, *y*). The spatial resolution of the temperature measurement is 3 $\upmu$m. The correlation between lifetime and temperature is presented in the supporting information (Sec. S2).

In a second step, the sample cell was filled with a suspension of fluorescently labeled G25 particles. The concentration was determined based on the fluorescence intensity. For excitation, we used a pulsed laser with a wavelength of 561 nm (Melles Griot, Green 85-YCA-015). We took first a reference image without a temperature gradient in order to determine the background intensity. After equilibrium of the concentration profile with an applied temperature gradient, a second image was taken. The concentration *c*(*x*, *y*, *z*) was calculated according to,

1 $$\begin{aligned} c(x,y,z) = c_{\mathrm {ref}} \times \frac{I(x,y,z)}{<I_{\mathrm {ref}}(x,y,z)>}, \end{aligned}$$

with the intensity *I*(*x*, *y*, *z*), the average intensity $<I_{\mathrm {ref}}(x,y,z)>$ and the concentration *c*(*x*, *y*, *z*). The reason of the averaged intensity $<I_{\mathrm {ref}}(x,y,z)>$ is described in the supporting information (Sec. S3). The spatial resolution of the concentration measurement is 1 $\upmu$m.

### Data evaluation

Based on the temperature *T*(*x*, *y*, *z*) and concentration *c*(*x*, *y*, *z*) profile, we determined the Soret coefficient. For a diluted solution with $c \ll 1$, the flux equation in the steady state is given by,

2 $$\begin{aligned} \nabla c =-c S_{\mathrm {T}} \nabla T, \end{aligned}$$

with the temperature *T* and the Soret coefficient $S_{\mathrm {T}}$. When we assume that the Soret coefficient is independent from the temperature and concentration in the measured condition, we can rewrite and integrate Eq.2 using $\nabla c = dc/dz$ and $\nabla T = dT/dz$ for a one-dimensional temperature profile in *z*-direction as follows:

3 $$\begin{aligned} \ln \frac{c(x,y,z)}{c(x,y,z_{\mathrm {ref}})} = S_{\mathrm {T}} (T(x,y,z_{\mathrm {ref}})-T(x,y,z)) \end{aligned}$$

Equation 3 shows that the logarithmic concentration ratio depends linearly on the temperature difference $T(x,y,z_{\mathrm {ref}})-T(x,y,z)$. The reference plane in this study is *z* = 10 $\upmu$m. The slope defines the Soret coefficient. For an 1D evaluation according to Eq. 3, we need to consider the temperature and concentration field around the microwire before establishing the data processing procedures. Figure 1b illustrates a possible thermophilic behavior around the heated microwire, implying that the particles move to the hot region. Since the temperature field is expected to show a radial dependence from the heated microwire, we infer that the 1D temperature distribution occurs at the center of microwire along the *z*-axis, so that Eq. 3 is valid.

**Fig. 2:**
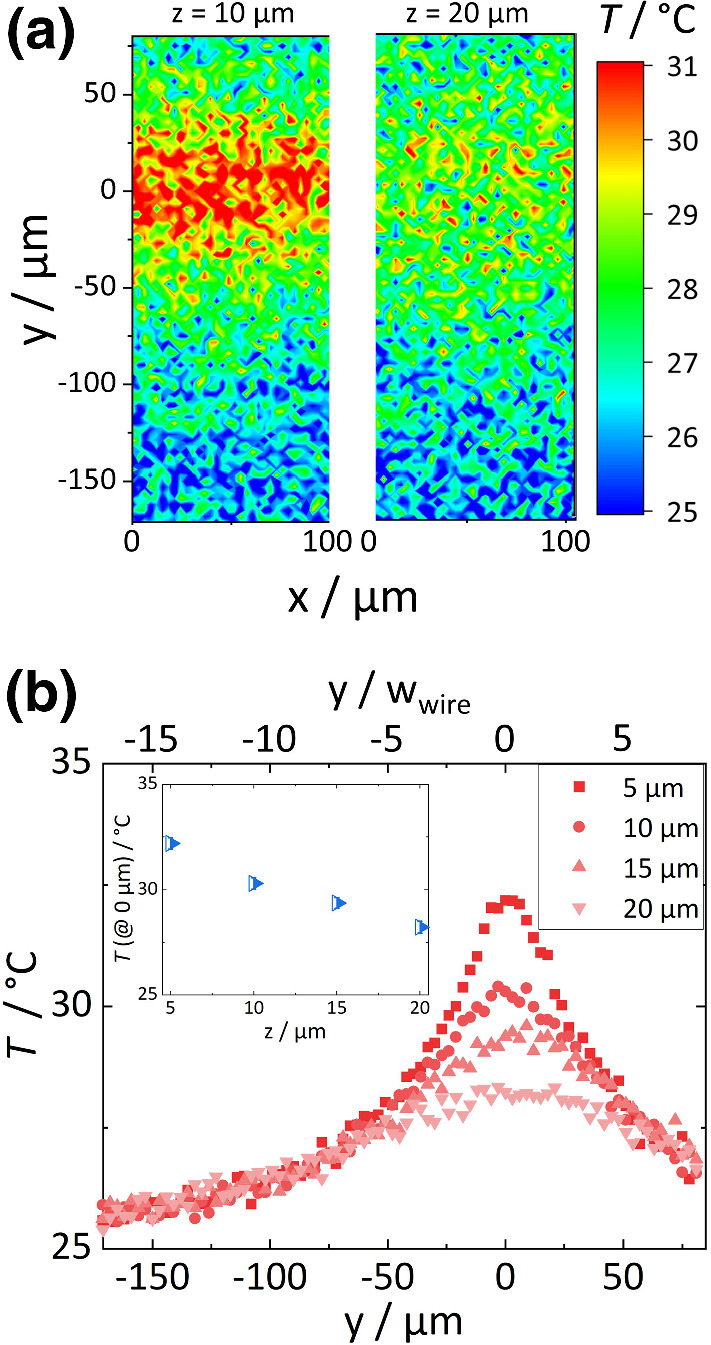
**a** Temperature distribution measured by fluorescence lifetime imaging microscope (FLIM) at a height of 10 $\upmu$m and 20 $\upmu$m above the chip surface. Graphs of **b** line-averaged temperature along the *x*-axis as a function of *y* at four different heights 5 $\upmu$m (square), 10 $\upmu$m (bullet), 15 $\upmu$m (triangle up) and 20 $\upmu$m (triangle down). Inset of **b** shows temperatures at the maximum (*y* = 0 $\upmu$m) as function of the height *z*

## Results

### Temperature profile

Figure 2a shows the temperature distribution at a height of $10~\upmu$m and 20 $\upmu$m when applying an electrical power of 1 W. When the distance from the heated microwire increases, the peak temperature decreases due to heat dissipation to the surrounding environment. While the temperature decreases in *y*-direction, the temperature gradient in *x*-direction is negligible. Note that an infinitely thin heated microwire should ideally induce a cylindrical temperature field in the *yz*-plane as sketched in Fig. 1. However, as shown in Fig. 2a, temperatures sufficiently far away from the microwire (*y* = −171 – −71 $\upmu$m) are overlapping for different heights. The prerequisites for cylindrical temperature profile are not fulfilled as the microwire width of 10 $\upmu$m is not sufficiently smaller than the chamber height of 50 $\upmu$m. Further discussion is described in the supporting information (S1). Additionally, the substrate is a silicon wafer with a high thermal conductivity (*k* $\sim$ 130 $\mathrm {W/mK}$) which dissipates the heat toward the surrounding, broadening the heated region. Another source of heat dissipation is the microscope objective, which is in thermal contact affecting the temperature field.

The derivation of Eq. 3 requires a one-dimensional temperature gradient. Looking at the temperature distribution displayed in Fig. 2, two areas with a one-dimensional temperature profile can be identified. The first area is between *y* = −171 – −71 $\upmu$m showing only a weak temperature dependence of $~0.01~\mathrm {K}/\upmu \mathrm {m}$, which is too weak to reliably determine the Soret coefficient. The second area corresponds to the maximum at *y* = 0 $\upmu$m. Here, the temperature varies between 32.2$^\circ$C to 28.2$^\circ$C in *z*-direction as shown in the inset of Fig. 2(b). This results in a temperature gradient of 0.26 $\mathrm {K}/\upmu \mathrm {m}$, which is typical for resistive heating [28] and higher than in other microscale devices for thermophoresis using a heating and a cooling channel [15–17]. Assuming convective effects to be small, this higher temperature gradient is advantageous to obtain more reliable Soret coefficients using Eq. 3.

**Fig. 3:**
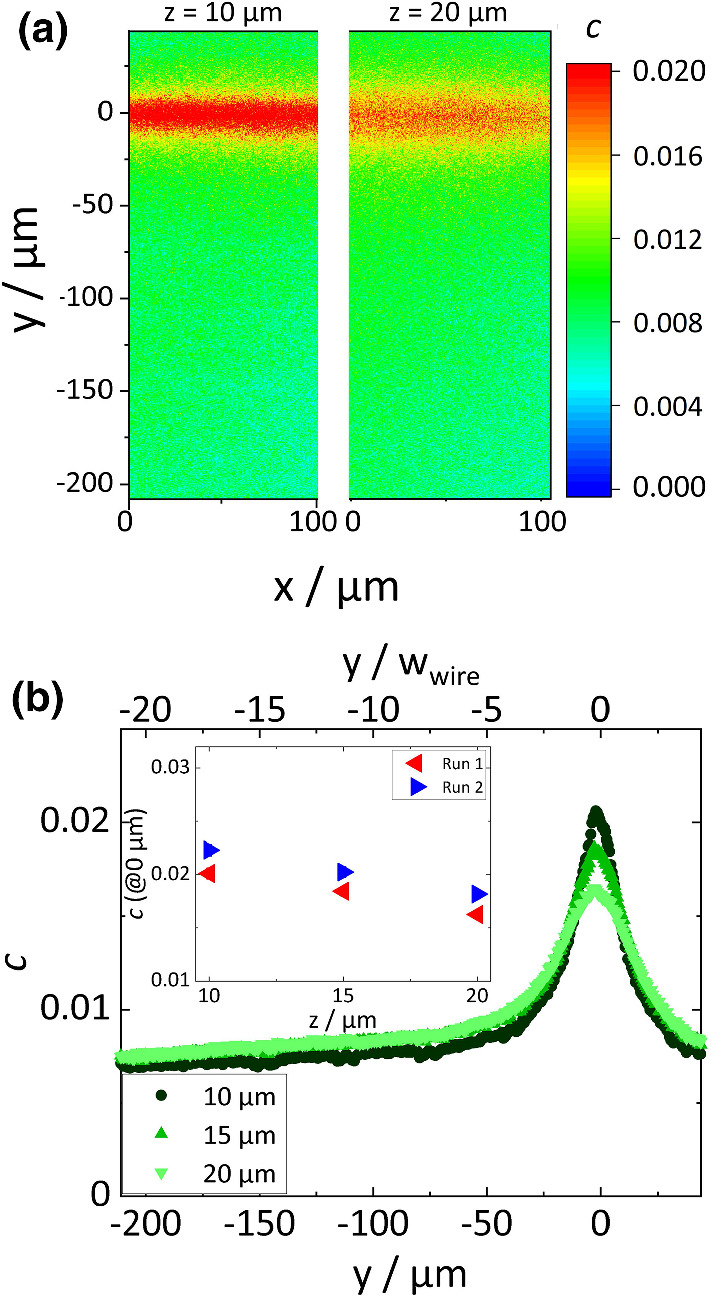
**a** Concentration distribution determined from fluorescence intensities at a height of 10 $\upmu$m, and 20 $\upmu$m above the chip surface. **b** Graph shows the line-averaged concentration (along *x*) as function of *y* at three different heights 10 $\upmu$m (light blue bullet), 15 $\upmu$m (medium blue triangle up) and 20 $\upmu$m (blue triangle down). The inset displays the maximum concentration of two different runs as function of height *z*

### Concentration profile

Figure 3a shows the concentration distribution at a height of *z* = 10 $\upmu$m and 20 $\upmu$m. Similar to the temperature profile, the maximum concentration decreases with increase in height, because the untreated particles are thermophilic and accumulate in the hot region [27]. Compared to the temperature distribution, the concentration profile is sharpened near the heated microwire, due to the logarithmic relation between concentration and temperature given by Eq. 3. Similar to the overlap of the temperature distributions, the concentration profiles far from the microwire (*y* = −171 – −71 $\upmu$m) overlap for different heights. The concentration profile is symmetric at the peak value and shows a one-dimensional concentration gradient along *z*.

Figure 3b presents concentrations averaged along the *x*-axis as function of *y*. In run 1, the maximum value is observed at 0 $\upmu$m. Note that each experiment is performed in two steps: first the temperature distribution is determined from a RhB solution and then the cell is cleaned thoroughly and refilled with the sample solution. Details of measurement procedures are described in the supporting information (Sec. S3).

The inset of Fig. 3b displays maximum values of the concentration for two runs. In both runs, 1 wt% G25 in solution from the supplier has been used. In a temperature gradient, the concentration varied from 1.6 to 2.0 wt% in run 1 and from 1.8 to 2.2 wt% in run 2. Even though the values were slightly different, the magnitude of the difference between 10 $\upmu$m and 20 $\upmu$m was similar along the *z*-direction.

**Fig. 4:**
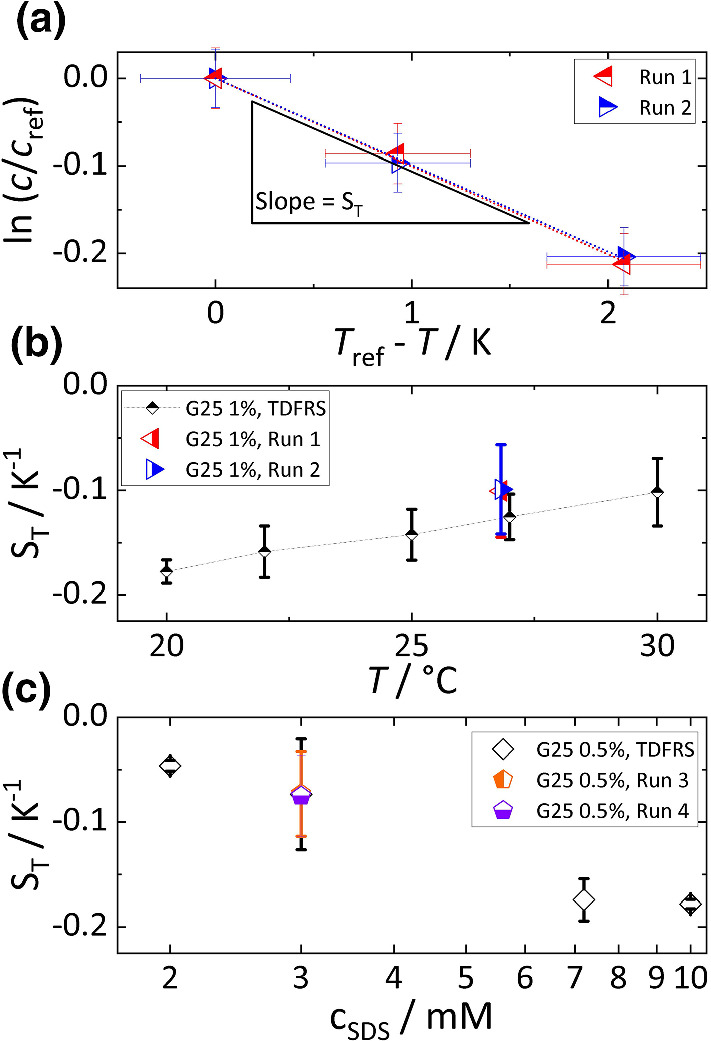
**a**  Soret coefficient Logarithmic concentration ratio as a function of temperature difference for runs 1 and 2. **b** Comparison of Soret coefficients of unwashed polystyrene particles (d = 25 nm, 1%) among validated TDFRS [27], run 1 and run 2. **c** Comparison of Soret coefficients of treated particles in 3 mM SDS surfactant between TDFRS [27], run 3 and run 4

### Evaluation of Soret coefficient

Using the concentration and temperature distributions at a height of 10 $\upmu$m as reference values, we show in Fig. 4a the logarithm of the concentration ratio as a function of the temperature difference for two different runs. According to Eq. 3, the Soret coefficient is determined by the slope. We find slopes of –0.10±0.04 in Run 1 and -0.10±0.04 in Run 2 including the errors of temperature and concentration. A detailed description of the uncertainty can be found in the supporting information (Sec. S4).

Figure 4b shows a comparison of Soret coefficients determined from the thermophoretic chip with literature data obtained in a validated TDFRS setup [27]. The averaged Soret coefficient at $T=(26.8 \pm 0.3)^\circ \mathrm {C}$ determined with the thermophoretic microchip in run 1 and 2 is $S_{\mathrm {T}}=($-$0.10 \pm 0.04) \mathrm {K}^{-1}$. This value is roughly 23% higher than $S_{\mathrm {T}}=(-0.13 \pm 0.02)\mathrm {K}^{-1}$ measured by TDFRS at 27$^\circ$C. Consequently, the values measured with our thermophoretic chips and those obtained by TDFRS agree within their uncertainties. Note that the same particle batch was used for the microchip and TDFRS experiments. Calculation of errors is described in the supporting information (Sec. S4).

In addition, we performed measurements of washed particles at a concentration of 0.5 wt% with 3 mM sodium dodecyl sulfate (SDS) for stabilization. The averaged Soret coefficient was $S_{\mathrm {T}}=(-0.07 \pm 0.04)\mathrm {K}^{-1}$ in runs 3 and 4, which agree with a recent TDFRS measurement of $S_{\mathrm {T}}=(-0.07 \pm 0.05)\mathrm {K}^{-1}$ of the same solution as shown in Fig. 4c. In the previous work [27], it was shown that the Soret coefficient is sensitive to the surfactant concentration. It is assumed that surfactant molecules adsorb at the surface of particles and alter the heat transport between the particle surface and the solvent, so that the thermophoretic behavior changes. Thus at low surfactant concentrations ($c \ll 0.1$ mM), a thermophobic behavior is observed, while particles at higher concentrations move to warmer regions. Note that SDS also induces a thermoelectric field influencing the thermophoretic movement of the charged micelles [29, 30]. The good agreement between $S_{\mathrm {T}}$ values measured with the thermophoretic microchip and the validated TDFRS method indicates that the surfactant concentration remains sufficiently constant although the thermophoretic microchip has a larger surface to volume ratio compared to the other experimental method. This ensures that the chip gives reproducible data even in the presence of surfactants.

## Conclusion

In summary, we introduced a thermophoretic chip for measuring Soret coefficients with a confocal microscope including a FLIM unit. Herein, the heat flow required to sustain a temperature gradient was generated via resistive heating of microstructured conductors on a chip’s surface. For observing the Soret coefficient, temperature and concentration fields were measured by FLIM and fluorescence intensity measurements, respectively. Exploiting the symmetry of temperature and concentration fields around the microwire, we recognized that one-dimensional temperature and concentration gradients can be found above the center of the microwire. Based on these one-dimensional gradients, a linear relation between the temperature difference and the logarithmic concentration ratio at two points can be derived as shown in Eq. 3. The slope of this linear expression gives the Soret coefficient. Measured values agree with Soret coefficients determined in a previous TDFRS experiment. Additionally, the chip reproduced the influence of SDS concentration on the Soret coefficient of particles.

This lab-on-a-chip device can be used in a biological/chemical laboratory as an analytical method to quantify the thermophoretic response of fluorescent proteins, biomolecules and colloids. With this chip, it will be possible to study changes of the thermophoretic response of proteins binding to a ligand molecule quantitatively. Due to the selective fluorescence, it will be possible to investigate systems in different buffers, as a function of ionic strength or in the presence of other compounds such as catalysts. The chip can also be used to study thermophobic and thermophilic macromolecules with a sufficiently high Soret coefficient of the order $S_{\mathrm {T}} \sim$ 0.1 $\mathrm {K}^{-1}$, but it will not be possible to study molecular solutions, which have a too low $S_{\mathrm {T}}$ of the order of ${\mathrm {10}}^{\mathrm {-3}}$ $\mathrm {K}^{-1}$. In the latter case, the measured concentration profile will be of the same order as the uncertainty of the concentration profile measurement. Additionally, as the chip provides rather large temperature gradients it might also be possible to test the linear response theory using colloidal spheres with varying diameter. In conclusion, we expect that this chip will be useful to investigate the fundamental mechanism of thermophoresis as well as to characterize chemical/biological colloids for pharmaceutical society.

## Supplementary Information

Below is the link to the electronic supplementary material.

Supplementary file 1 (pdf 998 KB)
