# Supplementary material for: Temperature profile characterization with fluorescence lifetime imaging microscopy in a thermophoretic chip
Source: Eur Phys J E Soft Matter. 2021 Oct 19;44(10):130. doi: 10.1140/epje/s10189-021-00133-7 (PMC8526468; doi:10.1140/epje/s10189-021-00133-7)
Supplement: Supplementary file 1 — (pdf 998 KB) [file 10189_2021_133_MOESM1_ESM.pdf]

SUPPORTING INFORMATION: Temperature profile characterization with fluorescence lifetime imaging microscopy in a thermophoretic chip

Namkyu Lee, Dzmitry Afanasenkau, Philipp Rinklin
Bernhard Wolfrum and Simone Wiegand

June 29, 2021

Contents

S1 Experimental set-up **S2**

S2 Calibration of rhodamine B for fluorescence lifetime imaging microscope (FLIM) **S3**

S3 Data analysis **S4**

S3.1 Reference concentration S4

S4 Error propagation **S5**

S1 Experimental set-up

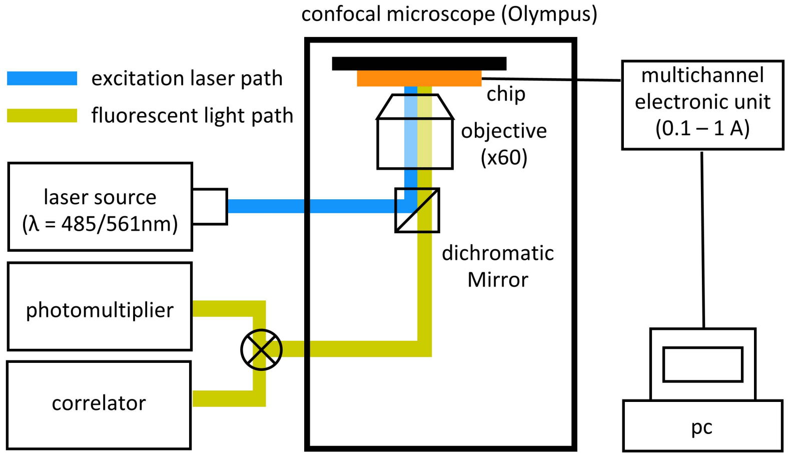

Figure S1: Schematic of the experimental set-up for measuring the Soret coefficient with the fabricated chip

The experimental set-up is shown in Fig. S1. The sample were investigated with an inverted confocal microscope (Olympus IX-71 with FV3-294 confocal unit) with a FLIM system (PicoQuant, Berlin, Germany). Lasers operating at a wavelength of 485 nm (Melles Griot, Blue LDH-P-C-485B) and 561 nm (Melles Griot, Green 85-YCA-015) are used for fluorescence lifetime and intensity measurements, respectively. The fluorescence lifetime was determined by a correlator and the fluorescence intensity was measured by a photomultiplier. The dichromatic mirror inside the microscope separates the excited laser and fluorescent light. The fluorescence light passed a long-pass emission filter at 500 nm (HQ 500 LP, Leica Microsystem GmbH, Wetzlar, Germany). The recorded data were evaluated by a commercial software (SymPhoTime provided by PicoQuant). We used an UIS2 (60x, immersion water) objective.

The lateral and axial the full width at the half maximum (FWHM) resolution of the confocal microscope can be estimated by [1].

$$\mathrm{FWHM}_{\mathrm{lateral}} = \frac{1}{\sqrt{1+\beta^2}} \cdot \frac{0.61 n \lambda_{\mathrm{ex}}}{NA} \tag{S1}$$

$$\mathrm{FWHM}_{\mathrm{axial}} = \frac{1}{\sqrt{1+\beta^2}} \cdot \frac{2 n \lambda_{\mathrm{ex}}}{NA^2}, \tag{S2}$$

with the refractive index n, the numerical aperture NA, the excitation wavelength λ_{ex}, the ratio between the excitation and detection wavelength ($\beta = \lambda_{\text{ex}}/\lambda_{\text{det}}$), respectively. With a NA=1.29 and assuming the refractive index of water for the diluted the solutions we find a lateral and axial resolution of 0.2 μm and 0.6 μm, respectively.

At the microscope, the encapsulated chip is installed in the inverted direction (i.e. the microwire surface is exposed toward the objective) to prevent free convection. The geometry of the chip results in a Rayleigh number of 700, which is significantly lower than 1700 [2]. Note, that the requirements for a cylindrical temperature profile around the wire are not met. This is due on the one hand to the finite width of the wire (10 μm), which is comparable to the height of the cell (50 μm), and on the other hand to the fact that due to the small size of the microchip there is coupling of the ambient temperature. Since it has been found that we only have a linear temperature profile directly above the wire, the finite width of the wire is even advantageous because over the entire width of the wire the temperature is constant. However, an experimental determination of the temperature profile is inevitable. The installed chip was connected to a multichannel electronic unit. The induced current is controlled with a connected power supply. The induced power is limited to 2 W.

S2 Calibration of rhodamine B for fluorescence lifetime imaging microscope (FLIM)

For fluorescence lifetime imaging microscope (FLIM), fluorescent dyes need to be calibrated for converting the fluorescence lifetime into a temperature. Rhodamine B (RhB) was used as fluorescent dye for FLIM to measure the temperature. The concentration of RhB solution was 0.3 mg/mL. For the calibration, we used an object slide with a cover glass containing the RhB solution sealed with sides and a thermistor attached at the other side of the object slide to probe the actual temperature. The assembled cover glass was installed in a thermostated holder regulated by a thermostat (ThermoHaake C25P, Thermoscientific, USA). Figure S2 shows measured fluorescence lifetimes as function of temperature. Our data compare well with literature data [3]. We used a 5th-order polynomial fit as a calibration curve to determine the temperature inside the chamber on the chip.

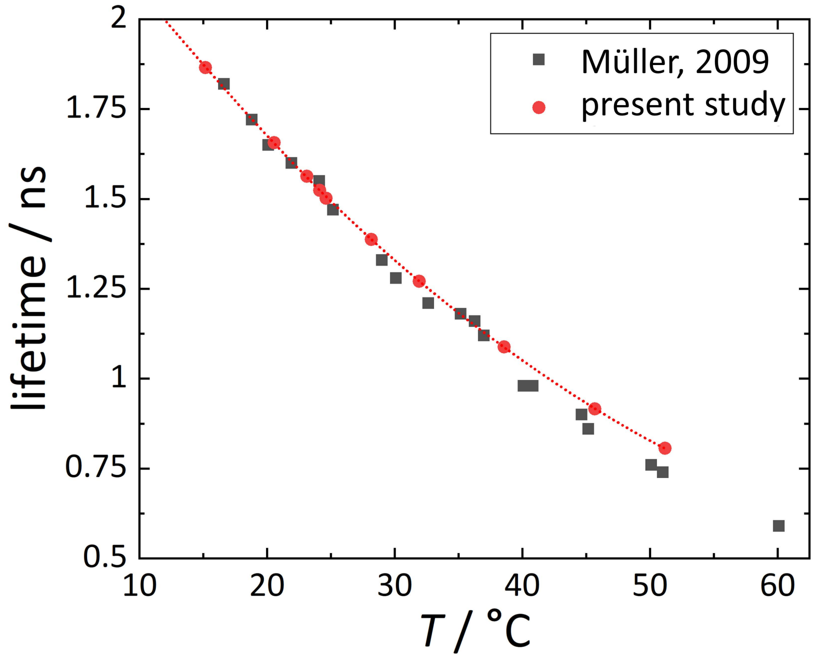

Figure S2: Measured lifetime of a RhB solution as function of temperature. Our data points (red bullets) are compared to data (black squares) by Müller *et al.* [3]. The solid line is a 5th-order polynomial fit used as calibration curve.

S3 Data analysis

S3.1 Reference concentration

Figure S3(a) shows a uniform temperature distribution along the y-axis at a height of $z = 10$ μm above the thermophoretic chip. The averaged temperature is 21.3°C and the deviation is 0.2°C. At the same time, the fluorescence intensity shows a shallow maximum (cf. Fig. S3(b)) probably caused by inhomogenous illuminations and/or reflections from the chip. In contrast to the inhomogenous intensity profile in the absence of a temperature gradient, the FLIM measurements are robust leading to a constant temperature profile across the chip.

Performing the data analysis as described in previous section, we can follow two routes to determine the Soret coefficient S_T using Eq.2.3. We can either normalize the concentration distribution by the average intensity $< I(x, y, z_{ref}) >_{x,y}$ or by $< I(x, y, z_{ref}) >_x$ explicitly considering the y-dependence. Depending on the average intensity, the determined S_T-values

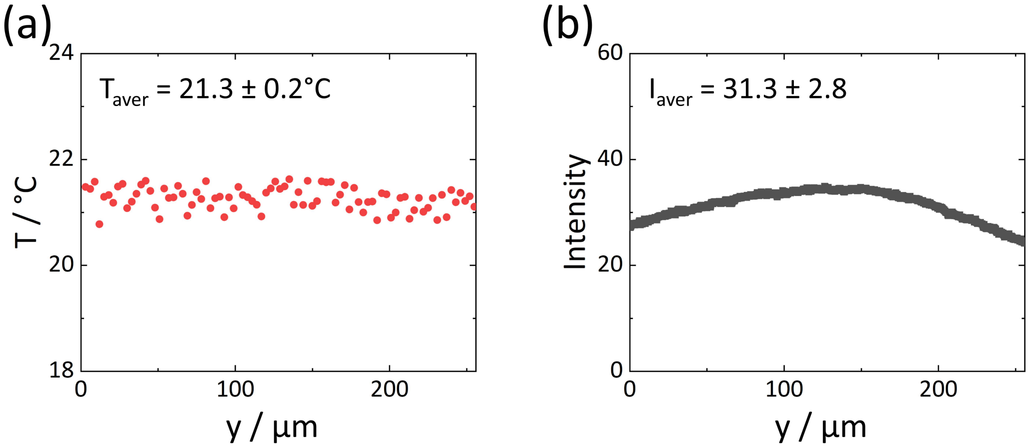

Figure S3: (a) Temperature and (b) intensity distribution along with Y-axis without heating the microwire.

above the microwire change by 7%, which is small compared to the standard deviation of 49% in run 1. Therefore, we used the average intensity $< I(x, y, z_{\mathrm{ref}}) >_{x,y}$ for the analysis.

S4 Error propagation

The Soret coefficient is given by the ratio of $\ln(c/c_{ref})$ and $T - T_{ref}$ according to Eq.(3) in the main manuscript. The uncertainty of the temperature is $\delta T = 0.27$ K and the relative concentration is $\delta c/c = 2.6\%$ in respect to the 95% confidence level [4]. The temperature uncertainty ranges from 0.25 K to 0.28 K. The relative concentration uncertainty ranges from 2.37% to 2.84%. Figure S4(a) shows $\ln(c/c_{ref})$ as function of $T_{ref} - T$ with the reference plane at z=10 μm. In run 1 a linear fit leads to S_{T} = -0.10 K^{-1}. In order to get the worst case errors for S_{T}, we performed linear fits with $(\Delta T \pm \delta(\Delta T)$, $\ln(c/c_{ref}) \pm \delta \ln(c/c_{ref}))$ to obtain the maximum and minimum value of S_{T}. This worst case analysis leads to a slope uncertainty of 0.044 K^{-1} in run 1 which is 44% of the Soret coefficient. In order to check the reliability of this analysis, we also calculate the slope uncertainty following the method by York (York method) [5] implemented in a commercial software [6]. This approach leads to a slope uncertainty of 0.036 K^{-1}. Taking the mean of uncertainties we conclude $S_{\mathrm{T}} = -0.10 \pm 0.04$ K^{-1}.

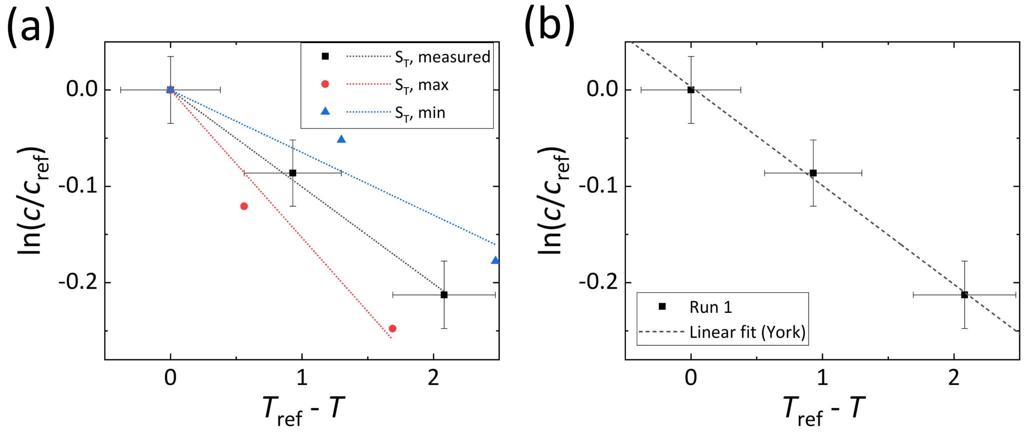

Figure S4: (a) Graph of measured, maximum (red) and minimum (blue) slope including the uncertainty of the temperature and concentration in run 1. The lines correspond to linear fits of the measured data points (black), the points with $(\Delta T - \delta(\Delta T), \ln(c/c_{ref}) - \delta \ln(c/c_{ref}))$ (red) and $(\Delta T + \delta(\Delta T), \ln(c/c_{ref}) + \delta \ln(c/c_{ref}))$ (blue). (b) Fitted graph including the temperature and concentration uncertainty following York method [5].

References

[1] Y. Kim, E. J. Lee, S. Roy, A. S. Sharbirin, L.-G. Ranz, Th. Dieing, and J. Kim. Measurement of lateral and axial resolution of confocal raman microscope using dispersed carbon nanotubes and suspended graphene. *Curr. Appl. Phys.*, 20:71–77, 2020.

[2] W. H. Reid and D. L. Harris. Some further results on the benard problem. *Phys. Fluids*, 1:102–110, 1958.

[3] C. B. Müller, K. Weiss, A. Loman, J. Enderlein, and W. Richtering. Remote temperature measurements in femto-liter volumes using dual-focus-fluorescence correlation spectroscopy. *Lab Chip*, 9(9):1248–1253, 2009.

[4] Robert J. Moffat. Describing the uncertainties in experimental results. *Exp. Therm. Fluid Sci.*, 1(1):3–17, 1988.

[5] D. York, N. M. Evensen, M. L. Martínez, and J. de Basabe Delgado. Unified equations for the slope, intercept, and standard errors of the best straight line. *Am. J. Phys.*, 72(3):367–375, 2004.

[6] OriginPro 2019. Originlab corporation. (Northampton, MA, USA).
